# Estimating usual intakes mainly affects the micronutrient distribution among infants, toddlers and pre-schoolers from the 2012 Mexican National Health and Nutrition Survey

**DOI:** 10.1017/S1368980015002311

**Published:** 2015-08-18

**Authors:** Carmen Piernas, Donna R Miles, Denise M Deming, Kathleen C Reidy, Barry M Popkin

**Affiliations:** 1 Department of Nutrition, Gillings School of Global Public Health, University of North Carolina at Chapel Hill, Carolina Population Center, 137 East Franklin Street, Room 6311, Chapel Hill, NC 27516-3997, USA; 2 Carolina Population Center, University of North Carolina at Chapel Hill, Chapel Hill, NC, USA; 3 Nestle Infant Nutrition Global R&D, Florham Park, NJ, USA

**Keywords:** Usual intake, Variability, Children, Nutrient adequacy

## Abstract

**Objective:**

To compare estimates from one day with usual intake estimates to evaluate how the adjustment for within-person variability affected nutrient intake and adequacy in Mexican children.

**Design:**

In order to obtain usual nutrient intakes, the National Cancer Institute’s method was used to correct the first 24 h dietary recall collected in the entire sample (*n* 2045) with a second 24 h recall collected in a sub-sample (*n* 178). We computed estimates of one-day and usual intakes of total energy, fat, Fe, Zn and Na.

**Setting:**

2012 Mexican National Health and Nutrition Survey.

**Subjects:**

A total of 2045 children were included: 0–5·9 months old (*n* 182), 6–11·9 months old (*n* 228), 12–23·9 months old (*n* 537) and 24–47·9 months old (*n* 1098). From these, 178 provided an additional dietary recall.

**Results:**

Although we found small or no differences in energy intake (kJ/d and kcal/d) between one-day *v*. usual intake means, the prevalence of inadequate and excessive energy intake decreased somewhat when using measures of usual intake relative to one day. Mean fat intake (g/d) was not different between one-day and usual intake among children >6 months old, but the prevalence of inadequate and excessive fat intake was overestimated among toddlers and pre-schoolers when using one-day intake (*P<*0·05). Compared with usual intake, estimates from one day yielded overestimated prevalences of inadequate micronutrient intakes but underestimated prevalences of excessive intakes among children aged >6 months.

**Conclusions:**

There was overall low variability in energy and fat intakes but higher for micronutrients. Because the usual intake distributions are narrower, the prevalence of inadequate/excessive intakes may be biased when estimating nutrient adequacy if one day of data is used.

Mexico is currently facing a high burden of nutrition-related non-communicable diseases including an increasing rate of childhood obesity, which poses important economic and long-term health consequences in the country^(^ ^1^ ^–^ ^5^ ^)^. Evaluation of current nutrient and energy intakes among Mexican infants and pre-schoolers is necessary to identify these major public health issues and help inform nutrition policies to promote the development of healthy eating patterns and decrease the burden of obesity and nutrition-related non-communicable diseases in Mexico.

Previous research on nutrient adequacy in young children has been useful to identify dietary patterns and food preferences that are established early in life that might help predict future eating habits^(^ ^6^ ^–^ ^8^ ^)^. In order to evaluate nutrient adequacy, measures of usual dietary intake are preferred to account for the day-to-day fluctuations in food consumption^(^ ^9^ ^,^ ^10^ ^)^. To date, only a few studies have investigated dietary adequacy of key nutrients and energy among Mexican children^(^ ^11^ ^–^ ^14^ ^)^, but none has accounted for the effect of within-person variability. Recent studies cast doubt on the utility of using a limited number of dietary recalls when evaluating nutrient adequacy^(^ ^9^ ^,^ ^10^ ^,^ ^15^ ^,^ ^16^ ^)^. Several strategies that incorporate a second day of intake while accounting for covariates that affect within-individual variation have shown an improved approximation to usual diet and more reliable estimates of the distribution of intakes^(^ ^10^ ^,^ ^15^ ^–^ ^18^ ^)^.

In children, within-person variability has been reported to be lower than between-person variability because of the lower diversity of foods eaten compared with adults^(^ ^9^ ^,^ ^19^ ^,^ ^20^ ^)^. This is especially true among infants and toddlers, who receive either breast milk or formula, and whose energy and nutrient requirements are supplied mainly by milk. At about 6 months of age, a few different foods are progressively introduced as the weaning process progresses so that within-person variability consequently increases^(^ ^9^ ^,^ ^21^ ^)^. Several studies regarding this issue have reported that, in general, fewer days are needed to assess energy and nutrient intakes among younger children compared with older children and adults^(^ ^9^ ^,^ ^10^ ^,^ ^22^ ^)^.

Most studies that include Mexican children have not addressed the problem of using one-day *v*. usual intake when determining the prevalence of inadequate or excessive intakes of important nutrients that are subject to within-individual day-to-day variation. We aimed to evaluate how adjustment for this variability affects energy intake and nutrient adequacy in a sample of Mexican infants, toddlers and pre-schoolers included in the 2012 National Health and Nutrition Survey. In order to obtain usual nutrient intakes, the National Cancer Institute’s (NCI) method was used to correct the first 24 h dietary recall collected in the entire sample with a second 24 h recall collected in a sub-sample. We computed estimates of one-day and usual intakes of total energy, fat, Fe, Zn and Na. These nutrients have critical roles in child growth^(^ ^23^ ^)^ and have also been found to be consumed in inadequate and/or excessive amounts among children of similar age in the USA^(^ ^6^ ^)^. In addition, inadequate intakes of these nutrients may indicate the coexistence of overnutrition (e.g. increased energy intake) and undernutrition (e.g. anaemia). There is evidence in Mexico both of a growing prevalence of child obesity and also a sub-population facing the double burden of under- and overnutrition and micronutrient deficiency^(^ ^24^ ^,^ ^25^ ^)^.

## Methods

### Study population: the Mexican National Health and Nutrition Survey 2012

The Mexican National Health and Nutrition Survey (NHNS) 2012 was conceived with the aim of characterizing the health and nutritional status of the Mexican population. The NHNS 2012 is a cross-sectional, probabilistic population-based survey with a multistage and stratified sampling, which represents the population of Mexico^(^ ^26^ ^)^. NHNS 2012 surveyed 50 528 Mexican households within thirty-two federal entities with a household response rate of 87 %. The NHNS 2012 sampling system included a proportional sample of both rural (population <2500 inhabitants) and urban (population >2500 inhabitants) areas that was drawn to be representative of four regional strata: (i) North (Baja California, Baja California Sur, Coahuila, Chihuahua, Durango, Nuevo León, Sonora, Tamaulipas); (ii) Center (Aguascalientes, Colima, Estado de México, Guanajuato, Jalisco, Michoacán, Morelos, Nayarit, Querétaro, San Luis Potosí, Sinaloa, Zacatecas); (iii) Mexico City; and (iv) South (Campeche, Chiapas, Guerrero, Hidalgo, Oaxaca, Puebla, Quintana Roo, Tabasco, Tlaxcala, Veracruz, Yucatán). Additional detailed description of the sampling procedures and survey methodology can be found elsewhere^(^ ^26^ ^)^.

### Dietary intake and nutrient data collection

Dietary intake was collected by trained interviewers using a 24 h recall in a stratified nationally representative sub-sample of the population (approximately one-sixth of the total population included in the NHNS 2012). In addition, a sub-sample was randomly selected by 6-month age strata during sampling development to report a second day of dietary intake. This second day was collected on a separate visit at least two days after the first recall. In the present study, from a total of 2057 infants, toddlers and pre-schoolers aged <4 years included in the NHNS 2012, 2045 had reliable dietary intake data from one 24 h recall of data and 178 had a second 24 h recall of dietary data.

For children <15 years of age, the main meal planner was asked to report all foods and beverages and the amount consumed of each food item for the previous 24 h period. In order to improve dietary recall, the NHNS 2012 implemented an automated five-step multiple-pass method based on different probes that ask for all foods and beverages consumed plus other typically forgotten foods, with a final probe for anything else that was consumed.

From the total sample of children <4 years of age with complete day 1 dietary data (*n* 2045), 351 reported any breast-feeding occasions. To estimate total daily volume from breast milk consistent with previous studies^(^ ^27^ ^–^ ^30^ ^)^, we assigned breast milk volumes using information on the child’s age and the total amount consumed of other milks (infant formula, cow’s milk and soya milk) if applicable. For both exclusively breast-fed and partially breast-fed children, the total daily volume from any breast-feeding or other milks assigned was 26·3745 fl oz (0·78 litres) for infants 0–5·9 months old and 20·2881 fl oz (0·60 litres) for infants 6–11·9 months old. For toddlers and pre-schoolers, the volume assigned per feeding occasion was 3 fl oz (88·72 ml) for children aged 12–17·9 months and 2 fl oz (59·15 ml) for those aged 18–41·9 months. For exclusively breast-fed children, the total volume of breast milk was equal to the total daily volume assigned for each age group. For mixed-fed children, the total volume from other milks or beverages was subtracted from the total volume assigned for each age group to estimate the total volume from breast milk. If the volume from other milks/beverages was greater than the total daily volume assigned, a volume of 3 fl oz was assigned to each breast-feeding occasion.

To capture energy, macro- and micronutrients in each food, beverage and breast milk, our study used the most recent food composition table, which was based on a combination of the chemical analyses conducted in Mexico on some unique foods and the food composition tables from the US Department of Agriculture’s Food and Nutrient Database for Dietary Studies (FNDDS)^(^ ^31^ ^,^ ^32^ ^)^. Macro- and micronutrients with critical roles in growth among children were selected for the present study, including total energy intake, total fat, Fe, Zn and Na^(^ ^23^ ^)^. Particularly, these nutrients have been found in previous research to be consumed in inadequate and/or excessive amounts among US infant, toddlers and pre-schoolers^(^ ^6^ ^)^.

### Nutrient adequacy assessment

The Institute of Medicine (IOM) Dietary Reference Intakes (DRI) have been widely used to evaluate nutrient adequacy and determine whether diets provide enough nutrients to ensure adequate growth without resulting in inadequate or excessive amounts to compromise health^(^ ^33^ ^,^ ^34^ ^)^. Means and percentiles (10, 25, 50, 75 and 90th) of reported one-day recall and usual intake (based on two-day recalls) were calculated for total daily energy, fat, Fe, Zn and Na. To investigate nutrient adequacies such as the prevalence of inadequate or excessive intakes, the proportion of participants above and below defined IOM DRI cut-off values was calculated^(^ ^33^ ^–^ ^35^ ^)^.

Total energy intakes by age group were evaluated against Estimated Energy Requirements (EER), which are helpful to understand if the population is in energy balance. To calculate EER, we used the equations provided by the IOM taking into account age, sex, body size and physical activity level (PAL)^(^ ^35^ ^)^. EER (sd) were calculated to estimate inadequate and excessive values by using the formula provided in Huang *et al*.^(^ ^36^ ^)^. Inadequate and excessive intake was classified as total energy intake less than 1·5 sd below the EER or more than 1·5 sd above the EER, respectively. This system has been used previously to identify under- and over-reporters^(^ ^18^ ^)^. For children >36 months of age, PAL were included in the equations to estimate EER, such that the lower bound for EER was calculated based on a sedentary PAL and the upper bound based on a very active PAL.

Adequate Intake (AI) values were utilized to evaluate inadequate intake of total fat for children <12 months of age, Fe and Zn for <6 months of age, and Na for all age groups. Although it is not appropriate to estimate nutrient adequacy percentage above and below the AI, if mean nutrient intake is at or above the AI value for a respective age group, then a low prevalence of inadequate intake can be assumed^(^ ^37^ ^)^. Acceptable Macronutrient Distribution Ranges (AMDR) were used to evaluate total fat intake for children 12–47·9 months of age. Intakes of Fe and Zn were evaluated against Estimated Average Requirements (EAR) for children 6–47·9 months of age. The percentage of the population with usual nutrient intakes lower than the EAR provides an estimate of those not meeting nutrient requirements^(^ ^37^ ^)^. Tolerable Upper Intake Levels (UL) are useful to study the percentage of the population with excessive intakes, which might increase adverse effects in the population^(^ ^33^ ^–^ ^35^ ^,^ ^37^ ^)^. To determine the proportion of children with excessive intakes, UL were used for Fe and Zn for children <12 months of age; whereas for children aged 12–47·9 months, UL were used for Fe, Zn and Na.

### Usual nutrient intake calculations

The present study implemented the NCI method to estimate the distribution of usual nutrient intakes using multiple recall dietary information^(^ ^15^ ^,^ ^17^ ^)^. The NCI method is useful to estimate the within- and between-person variances and correct for the high within-person day-to-day variation that is typically observed in 24 h recalls. The NCI method implements non-linear mixed regression models with a random effect to account for person-specific errors. The NCI method allows adjusting for important covariates that may influence variability (e.g. recall sequence, or weekend *v*. weekday recall) so that estimates will be adjusted for their effects, and also provides estimates for specific sub-populations. Another advantage of this method is that it is possible to estimate usual intake distributions even if repeated dietary data are available only for a sub-sample of the total population, such that individuals with only one 24 h recall will have predicted values of usual intake enhanced with the information from those with two 24 h recalls. We used the variance distribution from the 178 randomly selected duplicates within each age group to correct estimates of the larger sample.

For the current study, a one-part non-linear mixed model for repeated 24 h recalls was implemented using the NCI MIXTRAN macro^(^ ^15^ ^,^ ^38^ ^)^, with Box–Cox transformations and adjusting for weekend (including Friday) and recall sequence, by age group. The procedure was revised in collaboration with NCI programmers and statisticians to account for the structure of the Mexican survey design. Predicted usual intake values for each individual were estimated empirically using the NCI INDIVINT macro^(^ ^15^ ^,^ ^17^ ^,^ ^38^ ^)^, which performs adaptive Gaussian quadrature using the parameter estimates and linear predictor values from the mixed-effects model estimated by the NCI MIXTRAN macro. Although the covariate for weekend was included in the mixed-effects model, the NCI INDIVINT macro is unable to adjust for weekends at this time; thus, a weighted average of the weekday/weekend estimates was calculated for use in the NCI INDIVINT macro. Means and prevalence rates for inadequate and excessive usual intake values were compared between estimates from the NCI INDIVINT macro and those from the NCI DISTRIB macro, which allows adjusting for weekend/weekday but gives a population estimate instead of individual estimates, finding similar results between the two methods.

The NHNS 2012 uses a complex, multistage, probability design with 155 strata and 1596 primary sampling units (PSU; two to thirty-four PSU per stratum). Variance estimation was carried out via the BRR (Balanced Repeated Replication) technique with a Fay coefficient of 0·3^(^ ^39^ ^,^ ^40^ ^)^. Since the NCI BRR estimation method requires a stratified sample design with exactly two PSU in each stratum, two pseudo-PSU were created per stratum by randomly selecting half of the PSU in each stratum into one pseudo-PSU, with the other half, non-selected, combined in the second pseudo-PSU. Therefore, the 155 original strata were maintained with 310 pseudo-PSU, two per stratum.

### Statistical analysis

Participants of both sexes were grouped in four age groups: (i) infants aged 0–5·9 months; (ii) infants aged 6–11·9 months; (iii) toddlers aged 12–23·9 months; and (iv) pre-schoolers aged 24–47·9 months. The socio-economic status (SES) index was created using household demographic and SES variables, including characteristics of the head of the household, demographic household structure, home characteristics and appliances, household expenditure and level of marginalization of the geographic area^(^ ^41^ ^)^. The SES index was divided into tertiles and used as a proxy for low, medium and high SES in the present study. Participants’ weight and height were measured by trained staff using a standardized protocol^(^ ^26^ ^,^ ^42^ ^)^.

All analyses accounted for the complex survey design and sampling weights (dietary recall weights for day 1) using the statistical software package SAS version 9·3. Differences between the means of one-day *v*. usual intake for energy and nutrients were tested using Student’s *t* tests. Differences between the percentages above/below DRI cut-off values from one-day *v*. usual intake were tested using *χ* ^2^ tests. A two-sided *P<*0·05 was set to denote statistical significance.

## Results

Table 1 shows the demographic characteristics of the studied children. Our main sample included a higher proportion of toddlers (12–23·9 months) and pre-schoolers (24–47·9 months), from urban areas, and from central and southern geographic regions of Mexico. Compared with the main sample, the sub-sample of children with a second day of dietary data had similar distributions of age groups and other SES characteristics.

**Table 1:** Characteristics of Mexican infants, toddlers and pre-schoolers aged 0–47·9 months from the 2012 National Health and Nutrition Survey*

	Total sample (*n* 2045)		Sub-sample with second day of data (*n* 178)	
	*n*	%	*n*	%
Sex				
Male	1080	52	96	57
Female	965	48	82	43
Age group				
Infants 0–5·9 months	182	10	18	7
Infants 6–11·9 months	228	12	18	12
Toddlers 12–23·9 months	537	26	55	32
Pre-schoolers 24–47·9 months	1098	53	87	48
Area				
Urban	1247	70	117	69
Rural	798	30	61	31
Geographic area				
North	498	21	38	20
Central	729	32	65	32
Mexico City and metropolitan area	80	14	9	14
South	738	33	66	34
Socio-economic level				
Low	829	36	70	39
Medium	729	34	68	41
High	487	29	40	20
Feeding type				
Non-breast-fed	1694	83	146	89
Mixed-fed	308	15	25	9
Breast-fed	43	2	7	2

* Data presented are sample size and percentage; estimates were weighted to adjust for unequal probability of sampling.

EER and total daily energy intake distributions (one-day and usual intake; kJ/d and kcal/d) are displayed in Table 2. One-day means *v*. usual intake means of energy intake were not significantly different for any of the age groups except for toddlers, where the mean from one-day intake was slightly overestimated compared with the usual intake mean. The prevalence of inadequate intake of energy was overestimated when using measures of one day among infants 0–5·9 months old, toddlers 12–23·9 months old and pre-schoolers 24–47·9 months old, *P<*0·05; whereas the prevalence of excessive intake was overestimated among infants 6–11·9 months old and pre-schoolers 24–47·9 months old when using measures of one-day intake, *P<*0·05.

**Table 2:** Estimated energy requirements (EER), one-day and usual energy intake (EI) distributions of total daily energy (in kJ/d and kcal/d) for Mexican children aged 0–47·9 months (*n* 2045) by age group, 2012 National Health and Nutrition Survey*

					Percentiles											
	kJ/d		kcal/d		10th		25th		50th		75th		90th		Inadequate/excessive intake	
	Mean	se	Mean	se	kJ/d	kcal/d	kJ/d	kcal/d	kJ/d	kcal/d	kJ/d	kcal/d	kJ/d	kcal/d	%<−1·5 sd EER	% >+1·5 sd EER
0–5·9 months (*n* 182)																
EER	2475·3	17·8	591·6	4·3	2017·1	482·1	2251·6	538·2	2455·4	586·9	2677·6	640·0	2940·4	702·8		
One-day EI	2725·2	106·3	651·3	25·4	2100·7	502·1	2369·8	566·4	2375·2	567·7	2920·1	697·9	3942·6	942·3	8	20
Usual EI	2848·6	32·4	680·8	7·7	2607·4	623·2	2740·5	655·0	2743·4	655·7	2992·6	715·2	3404·9	813·8	4‡	25
6–11·9 months (*n* 228)																
EER	2880·2	27·7	688·4	6·6	2414·2	577·0	2569·2	614·0	2835·0	677·6	3160·7	755·4	3493·0	834·8		
One-day EI	3323·0	112·5	794·2	26·9	1818·0	434·5	2283·9	545·9	2798·9	669·0	4279·1	1022·7	5300·5	1266·8	5	24
Usual EI	3421·0	48·6	817·6	11·6	2754·8	658·4	3009·6	719·3	3221·9	770·0	3855·9	921·6	4321·4	1032·9	<1	14‡
12–23·9 months (*n* 537)																
EER	3641·6	32·8	870·4	7·8	2956·7	706·7	3276·9	783·2	3630·3	867·7	3905·2	933·4	4309·6	1030·0		
One-day EI	4807·0	138·0	1148·9	33·0	2227·0	532·3	2979·5	712·1	4261·6	1018·6	6364·9	1521·2	8243·1	1970·1	9	36
Usual EI	4638·5†	72·6	1108·6†	17·4	3465·0	828·2	3865·2	923·8	4532·3	1083·2	5378·7	1285·5	6040·4	1443·7	1‡	32
24–47·9 months (*n* 1098)																
EER (sedentary)	4593·1	17·8	1097·8	4·3	3998·9	955·8	4294·9	1026·5	4586·4	1096·2	4875·0	1165·2	5176·4	1237·2		
EER (very active)	5704·0	51·9	1363·3	12·4	3999·8	956·0	4410·0	1054·0	5611·5	1341·2	6988·5	1670·3	7342·8	1755·0		
One-day EI	5722·6	94·4	1367·7	22·6	2596·7	620·6	3701·8	884·8	5341·0	1276·5	7185·0	1717·3	8874·6	2121·1	9	18
Usual EI	5719·7	43·1	1367·0	10·3	4182·1	999·6	4861·2	1161·8	5684·3	1358·6	6454·8	1542·7	7109·7	1699·3	1‡	14‡

* EER were calculated for each age group using the Institute of Medicine equations^(^ ^35^ ^)^ and sd were calculated using the Huang formula^(^ ^36^ ^)^. Prevalence of inadequate total energy intake was calculated as<1·5 sd below EER; excessive energy intake was calculated as >1·5 sd above EER. For children 36–47·9 months of age, physical activity levels were included in the equations to estimate EER, so that the lower bound for EER was calculated using a sedentary physical activity level and the upper bound using a very active sedentary physical activity level.

† Significantly different between one-day *v*. usual intake, *P*<0·05 (Student’s *t* test).

‡ Significantly different between one-day *v*. usual intake, *P<*0·05 (*χ* ^2^ test).

One-day and usual intake distributions of total daily fat (g/d and percentage of total daily energy), Fe (mg/d), Zn (mg/d) and Na (mg/d) are reported for each age group in Tables 3 and 4. Overall, the mean total fat intake (g/d) was not significantly different when using one-day or usual intake except for infants aged 0–5·9 months, where the mean of one day was underestimated. The percentage of energy from fat was higher among infants aged 0–5·9 months and pre-schoolers when using measures of usual intake, *P<*0·05. The proportion of toddlers and pre-schoolers with inadequate or excessive fat intakes was overestimated when using measures of one day, *P<*0·05.

**Table 3:** One-day *v*. usual intake distributions of total fat, iron, zinc and sodium for Mexican infants aged 0–5·9 months (*n* 182) and 6–11·9 months (*n* 228), 2012 National Health and Nutrition Survey

	DRI*		Intake		Percentiles					Inadequate/excessive intake	
	EAR/AI	UL	Mean	se	10th	25th	50th	75th	90th	%<EAR/AI	% >UL
Infants 0–5·9 months											
Fat (g/d)											
One-day	31	–	35·1	1·3	16·9	31·5	35·4	35·7	46·1	–	–
Usual intake			39·6†	0·4	31·8	38·8	40·4	40·7	45·0	–	–
Fat (% of total daily energy)											
One-day	–	–	31·9	2·0	0·0	0·0	44·2	50·6	54·2	–	–
Usual intake			52·6†	0·3	46·1	50·3	53·7	55·5	55·5	–	–
Fe (mg/d)											
One-day	0·27	40	4·9	0·6	0·1	0·2	1·8	6·8	13·7	–	1
Usual intake			3·2†	0·2	0·9	1·0	2·3	4·6	6·9	–	<1
Zn (mg/d)											
One-day	2	4	3·7	0·3	1·2	1·3	2·3	4·3	7·9	–	30
Usual intake			3·2	0·1	2·2	2·3	2·9	3·7	4·8	–	21‡
Na (mg/d)											
One-day	120	–	183·2	22·1	0·0	0·4	87·6	242·2	427·1	–	–
Usual intake			172·2	8·2	28·9	50·4	171·0	250·0	312·0	–	–
Infants 6–11·9 months											
Fat (g/d)											
One-day	30	–	34·2	1·1	13·9	26·5	29·9	42·1	55·1	–	–
Usual intake			33·8	0·4	25·2	31·2	33·0	36·8	42·1	–	–
Fat (% of total daily energy)											
One-day	–	–	38·4	0·6	23·6	33·1	39·5	45·6	49·6	–	–
Usual intake			37·4	0·2	31·6	35·3	38·0	40·0	41·7	–	–
Fe (mg/d)											
One-day	6·9	40	6·8	0·4	0·9	1·5	3·1	10·0	16·1	63	1
Usual intake			6·3	0·2	2·8	3·4	4·8	8·8	11·3	61	<1
Zn (mg/d)											
One-day	2·5	5	5·4	0·3	1·3	2·0	3·5	6·4	11·0	32	42
Usual intake			5·3	0·1	3·3	4·0	4·9	6·2	7·5	<1†	48‡
Na (mg/d)											
One-day	370	–	710·0	41·5	67·1	228·5	476·3	836·7	1532·4	–	–
Usual intake			711·4	18·1	360·1	522·3	686·7	834·2	1098·6	–	–

DRI, Dietary Reference Intake; EAR, Estimated Average Requirement; AI, Adequate Intake; UL, Tolerable Upper Intake Level.

* For infants aged 0–5·9 months, AI were used for fat (g/d), Fe, Zn and Na; UL were used for Fe and Zn. For infants aged 6–11·9 months, EAR were used for Fe and Zn; AI were used for fat (g/d) and Na; UL were used for Fe and Zn. The dash symbol indicates that DRI is not available and the percentage with inadequate/excessive intakes cannot be estimated.

† Significantly different between one-day *v*. usual intake, *P<*0·05 (Student’s *t* test)

‡ Significantly different between one-day *v*. usual intake, *P<*0·05 (*χ* ^2^ test).

**Table 4:** One-day *v*. usual intake distributions of total fat, iron, zinc and sodium for Mexican toddlers aged 12–23·9 months (*n* 537) and pre-schoolers aged 24–47·9 months (*n* 1098), 2012 National Health and Nutrition Survey

	DRI*		Intake		Percentiles					Inadequate/excessive intake	
	AMDR/EAR/AI	UL	Mean	se	10th	25th	50th	75th	90th	%<AMDR/EAR/AI	% >AMDR/UL
Toddlers 12–23·9 months											
Fat (g/d)											
One-day	–	–	42·1	1·4	16·0	24·1	36·1	54·6	71·2	–	–
Usual intake			41·3	0·7	30·8	34·8	40·1	46·9	53·6	–	–
Fat (% of total daily energy)											
One-day	30–40	–	33·3	0·5	19·4	27·3	34·0	39·8	44·7	33	24
Usual intake			33·9	0·2	28·1	31·6	34·3	36·4	38·5	17‡	4‡
Fe (mg/d)											
One-day	3	40	8·8	0·5	2·3	3·7	7·0	11·6	17·7	18	<1
Usual intake			8·5	0·2	4·5	5·8	8·0	10·3	13·3	1‡	<1
Zn (mg/d)											
One-day	2·5	7	6·8	0·2	2·2	3·8	6·3	9·0	11·9	13	42
Usual intake			7·0	0·1	4·7	5·7	7·0	8·2	9·1	<1‡	51‡
Na (mg/d)											
One-day	1000	1500	1378·4	63·6	392·6	666·3	1084·0	1682·5	2425·2	–	32
Usual intake			1340·3	19·3	883·6	1049·3	1294·7	1529·1	1791·1	–	30
Pre-schoolers 24–47·9 months											
Fat (g/d)											
One-day	–	–	50·6	1·2	16·7	29·5	44·0	64·8	87·8	–	–
Usual intake			51·0	0·5	36·6	43·7	50·1	57·6	64·9	–	–
Fat (% of total daily energy)											
One-day	30–40	–	32·8	0·5	18·1	25·9	33·3	40·4	45·1	36	26
Usual intake			33·7†	0·2	28·0	31·6	34·3	36·5	38·4	17‡	5‡
Fe (mg/d)											
One-day	3	40	10·1	0·2	3·5	5·2	8·4	13·4	18·7	7	1
Usual intake			10·9†	0·1	6·8	8·2	10·4	13·2	15·7	1‡	<1
Zn (mg/d)											
One-day	2·5	7	7·9	0·2	3·1	4·6	7·0	10·1	13·7	6	48
Usual intake			8·5†	0·1	6·1	7·1	8·3	9·7	11·0	<1‡	73‡
Na (mg/d)											
One-day	1000	1500	1820·4	54·1	583·9	910·6	1473·9	2234·2	3268·0	–	47
Usual intake			1811·2	20·8	1225·5	1447·7	1741·1	2056·0	2428·5	–	69‡

DRI, Dietary Reference Intake; AMDR, Acceptable Macronutrient Distribution Range; EAR, Estimated Average Requirement; AI, Adequate Intake; UL, Tolerable Upper Intake Level.

* AMDR were used for total daily fat (% of total daily energy); EAR were used for Fe and Zn; AI were used for Na; UL were used for Fe, Zn and Na. The dash symbol indicates that DRI is not available and the percentage with inadequate/excessive intakes cannot be estimated.

† Significantly different between one-day *v*. usual intake, *P<*0·05 (Student’s *t* test).

‡ Significantly different between one-day *v*. usual intake, *P<*0·05 (*χ* ^2^ test).

Estimates of the mean intakes and the distributions of micronutrients were slightly more affected by day-to-day variation among infants aged 0–5·9 months and pre-schoolers (Tables 3 and 4). Mean Fe and Zn intakes were not different when using one-day *v*. usual intakes among infants 6–11·9 months old and toddlers 12–23·9 months old. The percentage above the UL for Fe was similar across all ages, whereas the percentage above the UL for Zn was significantly underestimated among infants aged 6–11·9 months, toddlers and pre-schoolers but overestimated among infants aged 0–5·9 months when using one day of intake, *P<*0·05. The proportion of children with inadequate Fe and Zn intakes was significantly overestimated among toddlers and pre-schoolers when using one-day intake. Among infants, the proportion with inadequate intake of Fe was not different with the two methods, but for Zn the proportion with inadequate intake was overestimated when using one-day intake. Although mean Na intake was not significantly different between one-day *v*. usual intake for any age group, the prevalence of excessive Na intake was significantly underestimated among pre-schoolers aged 24–47·9 months when using one day of intake, *P<*0·05.

## Discussion

The present study is one of the few that included a sample of the youngest age groups and implemented statistical methods to investigate how adjustment for within-individual day-to-day variation affected nutrient intake and dietary adequacy of selected macro- and micronutrients in a sample of Mexican children <4 years of age. The NCI method was implemented to correct for within-individual day-to-day variation for a better approximation of usual intake. Overall, after including a second day of dietary intake, a reduced variability was reported for all estimates as shown by the narrower tails of the distributions across all age groups.

We found an overall low variability in energy intake across all ages, although the prevalence of inadequate or excessive intake of energy decreased somewhat when using measures of usual intake relative to one day. Variability in the mean fat intake was also low except for infants aged 0–5·9 months, but the prevalence of inadequate and excessive intakes as measured by the AMDR was overestimated among toddlers and pre-schoolers when using one-day intake. Regarding micronutrient intakes, we found small or no differences in the mean Fe and Zn intakes and no differences in the mean Na intake for any age group. Previous literature has reported a lower variability in energy and nutrient intakes among children compared with adults and among younger children compared with older children^(^ ^9^ ^,^ ^10^ ^,^ ^19^ ^–^ ^22^ ^,^ ^43^ ^)^. In general, about four to six 24 h recalls have been reported as optimal for studying most nutrients and food groups^(^ ^44^ ^)^. Among older children and adolescents, approximately six to nine recalls are needed to obtain accurate nutrient intakes with a reasonable participant burden^(^ ^45^ ^)^, whereas two to five recalls are needed among younger children <2 years of age because between-subject variability is greater than within-subject variability in this age group^(^ ^9^ ^)^. However, our study showed higher variability in fat (g/d and percentage of total daily energy) and Fe intakes among younger infants 0–5·9 months old, which might reflect an increased day-to-day variability in the number of breast-feeding occasions in this age group. We also found a slightly higher variability in fat, Fe and Zn intakes among pre-schoolers. Although significant, the small differences seen in pre-schoolers may be a function of a higher sample size but have little public health significance. Na intake showed low variability in all age groups as no differences were found between estimates of one-day *v*. usual intakes.

We showed small or no differences in the mean total energy (kJ/d) and fat intakes (g/d) when using one-day *v*. usual intake data. Overall, total daily energy and fat intakes are moderately affected by day-to-day variation because energy and macronutrient intakes are generally well regulated physiologically and because macronutrients come from a wide variety of foods^(^ ^20^ ^)^. However, micronutrients are usually concentrated in certain foods, so mean intakes might be very high or very low depending on food choices, contributing to a higher between-day variation^(^ ^9^ ^,^ ^10^ ^,^ ^19^ ^,^ ^46^ ^)^. This issue of a higher variability in micronutrient intake might result even more problematic in studies of dietary adequacy in developing countries, where food choices might be associated with income or even seasonality if transportation and preservation of foods are limited^(^ ^22^ ^,^ ^47^ ^)^. Our sample was mainly from urban areas, so we can expect that a high proportion of the variation in micronutrient intakes was largely due to variation in daily food selection and choices.

Even though measures of one or limited days of dietary intake can provide accurate estimates of mean nutrient intakes at the population level, the effect of day-to-day variation is mainly reflected by an overestimated sd. In consequence, estimates of the prevalence of inadequate or excessive intake can be biased if only one day of intake is available^(^ ^19^ ^,^ ^46^ ^)^. Compared with usual intake, estimates from one day in our study tended to yield overestimated prevalences of inadequate micronutrient intakes but underestimated prevalences of excessive micronutrient intakes among children aged >6 months, with the exception of Fe among children aged 6–11 months. This may be due to the relatively few different foods and few foods that are good sources of Fe consumed by children in this age group.

We implemented the NCI method to estimate the distribution of usual nutrient intake using dietary information of a second 24 h recall collected on a sub-sample of participants^(^ ^15^ ^,^ ^17^ ^)^. The NCI method is useful to correct for the high within-person day-to-day variation intrinsic to 24 h recalls and allows adjusting for covariates that influence that variability. However, our method might still have certain limitations. Originally, the NCI method was specifically developed to be applied to the US Department of Agriculture’s National Health and Nutrition Examination Surveys (NHANES), with its unique PSU, strata system and weighting factors; and where the majority of the participants usually provide two days of dietary intake. In contrast, our survey had a different PSU scheme and included a second day of intake only for approximately 10 % of the total sample. With a small sub-sample with a second day of dietary intake, we might not have a complete approximation to the within-person variance. Additionally, we had to generate ‘pseudo-PSU’ to accommodate the NCI method requirement of two PSU per stratum, so these methodological and technical challenges need to be taken into account when interpreting our results.

Additional limitations related to the design of the NHNS 2012 might also affect our results.

The NHNS 2012 is a cross-sectional observational data set and our analysis used self-reported intake data over one or two 24 h periods of time. Self-reported intake might be affected by recall bias and/or reporting errors from the proxy recall in our sample of children. Such measurement error might be randomly distributed across the different age subgroups or might affect certain sub-populations systematically (i.e. dietary recall might be easier for children <1 year of age because food variability is usually lower compared with older children). Also, accuracy of dietary reporting might differ by SES, literacy of the proxy meal planner and/or the child‘s nutritional status. Despite these limitations, the NHNS 2012 is the most comprehensive nationally representative data set for studying dietary intake and nutrient adequacy in the Mexican population. Additionally, we used the IOM DRI to study nutrient adequacy in our Mexican population. It should be noted that, for many nutrients among infants 0–11·9 months old, EAR are not available and only AI have been established. AI reflects the mean intake of a healthy population, so the proportion of the population with inadequate intake cannot be calculated based on AI^(^ ^37^ ^)^.

It is essential to understand the importance of using one or more days of dietary intake when investigating nutrient adequacies in nutritional epidemiology studies. In general, measures of usual intake are needed to study diet–disease relationships and in clinical settings when designing appropriate interventions. However, there is a trade-off between achieving accurate measures of usual intake and the burden for both the participant and investigator, which is particularly problematic in large epidemiological studies. Additionally, data collection is especially burdensome in infants and children because the investigator needs to rely on parental recall at a time when child feeding may be stressful.

## Conclusions

Although measures of usual intake may be desirable, our study showed an overall low variability in energy and fat when using one-day *v*. usual intakes. Since the usual intake distributions are narrower, the prevalence of inadequate or excessive intake may be biased when estimating nutrient adequacies if one day of dietary data is used. Our study has implications for future research involving very young children, or even in young populations from other countries that are currently undergoing a nutrition transition and facing both rising obesity and the double burden of under-, overnutrition and/or micronutrient deficiency. Our results are also valuable for future studies involving Mexican children as we highlighted the importance of repeated 24 h recalls in order to adjust for intra-individual variability to study energy intake and dietary adequacy of critical nutrients that are related to growth. Further research should investigate if there are systematic differences across diverse sub-populations, especially considering SES groups or geographical regions of Mexico.
